# Caregiver Experiences, Healthcare Provider Perspectives and Child Outcomes with Virtual Care in a Neonatal Neurodevelopmental Follow-Up Clinic: A Mixed-Methods Study

**DOI:** 10.3390/children11111272

**Published:** 2024-10-22

**Authors:** Kamini Raghuram, Hayle Noh, Seungwoo Lee, Nicole Look Hong, Edmond Kelly, Vibhuti Shah

**Affiliations:** 1 Department of Paediatrics, Mount Sinai Hospital, Toronto, ON M5G 1X5, Canada; kamini.raghuram@sinaihealth.ca (K.R.); edmond.kelly@sinaihealth.ca (E.K.); 2 Global Health Program, McMaster University, Hamilton, ON L8S 4L8, Canada; 3 Maternal-Infant Care Research Centre, Mount Sinai Hospital, Toronto, ON M5G 1X5, Canada; seungwoo.lee@sinaihealth.ca; 4 Department of Surgery, Sunnybrook Health Sciences Centre, Toronto, ON M4N 3M5, Canada; 5 Institute of Health Policy, Management and Evaluation, University of Toronto, Toronto, ON M5S 1A1, Canada

**Keywords:** virtual care, neonatal follow-up, health services

## Abstract

Background: Caregiver and healthcare provider perspectives of virtual care have not been explored in depth in the literature for neonatal follow-up clinics. Our objective was to evaluate caregivers’ and healthcare providers’ perspectives and compare neurodevelopmental outcomes of preterm neonates before and after implementing virtual care during the SARS-CoV-2 pandemic. Methods: Semi-structured interviews were conducted with families and healthcare providers, designed and analyzed using phenomenological qualitative methods. A retrospective cohort study was conducted to evaluate and compare neurodevelopmental characteristics of two preterm cohorts, one before (“in-person”) and after (“virtual”) virtual care. Results: Three themes were identified: increased confidence in in-person assessments, adequate delivery of information using virtual platforms and a preference for specialized care through the neonatal follow-up clinic. A total of 252 infants born preterm, 104 infants in the in-person group and 148 infants in the virtual group, were included in the study. The adjusted odds ratio (aOR) of cerebral palsy was lower when virtual care was used compared to in-person assessments (aOR = 0.11, 95% CI 0.01–0.98) while the adjusted odds of cognitive delay measured by in-person standardized testing were higher (aOR = 2.78, 95% CI 1.25–6.19). Conclusions: Caregivers and healthcare providers prefer in-person assessments for comprehensive developmental support. It may be more challenging to detect subtle cognitive differences using caregiver-reported measures. Cerebral palsy may be missed when assessments are completed virtually.

## 1. Introduction

During the SARS-CoV-2 pandemic, all non-urgent in-person care was cancelled, including neonatal neurodevelopmental follow-up (NNFU). Virtual care then became necessary to deliver ongoing care to high-risk infants, primarily those born very preterm <29 weeks gestational age and those with high-risk conditions such as hypoxic-ischemic encephalopathy or antenatally diagnosed neuroanatomical abnormalities, from the Neonatal Intensive Care Unit (NICU) [1,2,3]. As a result of this rapid transition to virtual care, many clinics have not had the opportunity to evaluate patient experiences and create family-centred programs [4].

While telemedicine has existed since the 1950s [5], its use was propelled forward in a number of clinical contexts after the advancement of communication technologies in the 1990s and 2000s and most recently with the SARS-CoV-2 pandemic [6]. Telemedicine used for regular follow-up visits in infants who were recently discharged from the NICU was associated with a decrease in emergency room visits and may help ease the transition between the NICU and home [7,8]. Telehealth has also been used for management of behavioural issues, access to expert care for Attention Deficit Hyperactivity Disorder (ADHD), [9] autism diagnostic assessments [10], and for the early identification of cerebral palsy [11]. The use of such programs has demonstrated improved follow-up rates [12] and high patient satisfaction [13]. Thus, it is likely that there are enduring advantages to using telemedicine in neonatal follow-up clinics.

Prior to its integration, it is imperative that we understand caregiver and healthcare provider perspectives, develop and implement normative standards of practice and create an organizational structure to support the use of these technologies [12,14]. To that end, the following study aims to characterize the perspectives of families and healthcare providers of virtual care in NNFU. Using qualitative methodology, we aimed to identify themes that were congruent and incongruent between caregivers and healthcare providers regarding virtual care delivery. These themes could then be used to implement changes necessary to improve its quality and allow for permanent integration into routine care. To further evaluate the impact that virtual care has on being able to effectively assess neurodevelopment in children born preterm, we aimed to compare neurodevelopmental outcomes before and after virtual care was implemented and thus, the ability for developmental delays to be detected using virtual care. The following study includes the methodology used in both the qualitative and quantitative parts of the study, the results of the caregiver and healthcare provider interviews, a comparison between the neurodevelopmental outcomes of preterm children before and after the implementation of routine virtual care and a discussion around common themes surrounding virtual care perspectives and next steps in the use of virtual care in neonatal neurodevelopmental follow-up clinics.

## 2. Materials and Methods

### 2.1. Setting and Study Design

This was a mixed-methods study and included a qualitative and quantitative component. The qualitative component focused on caregiver and healthcare provider perspectives of virtual care in NNFU. The quantitative component was a retrospective cohort study evaluating neurodevelopmental outcomes before and after virtual care was implemented. The study was conducted at a large tertiary NNFU (Mount Sinai Hospital) in Toronto, Canada. Ethics approval was obtained prior to the initiation of the study (Research Ethics Board approval #22-0010-E).

### 2.2. Study Participants, Clinical Data Collection and Outcomes

#### 2.2.1. Qualitative Study

For the qualitative study, purposeful sampling of families and healthcare providers at NNFU was conducted. The phenomenon of interest was the implementation of virtual care, including audio- and video-conferencing platforms used for the purposes of clinical assessment during the SARS-CoV-2 pandemic in the context of the NNFU. The emphasis of the study was to describe the perspectives of caregivers and healthcare providers who were exposed to virtual care compared to in-person care. Descriptive characteristics of the infants were collected from patient charts.

Families of high-risk infants attending neonatal follow-up who had their detailed developmental assessment completed virtually versus those who had it completed in person, who spoke English and had attended at least two appointments were included in the study. Healthcare professionals who took part in both types of visits and spoke in English were included. Target groups included those who had worked in the clinic prior to the start of the pandemic or only worked after the pandemic started.

Informed consent was obtained prospectively prior to the initiation of the interview from both families and healthcare providers. A semi-structured one-on-one interview with study personnel was used to collect data from families and healthcare providers who provided consent. The interview was designed based on previous literature describing (a) ideal virtual care in outpatient practice [6] and (b) ideal virtual care for neonatal follow-up [7]. Caregivers were recruited and interviewed by one author (HN), who was not involved in clinical care, immediately following their most recent clinic visit via telephone or video-conferencing, whichever was preferred for the family. Families did not receive a guide for interviews, but a semi-structured guide was used by the interviewer to conduct the interviews. Transcripts of the interviews were generated from recordings; these were not given to families after the interview was complete and their feedback was not sought as part of this study. The interview questions are provided in the Supplementary Materials (Material S1).

#### 2.2.2. Quantitative Study

For the quantitative study, we included preterm infants [gestational age (GA) < 37 weeks] born between November 2017 and December 2019 who had attended at least one NNFU visit. Children who attended their 18–24-month visit when only in-person care was available (before 1 March 2020) were considered to be in the “in-person” cohort. Following a washout period of 4 months until 1 July 2020, children who attended their 18–24-month visit after this time were considered to be in the “virtual” cohort. During the pandemic, virtual visits were conducted for all children. If a child was identified as having a developmental concern based on virtual assessment, an in-person assessment was completed, including standardized developmental testing using the Bayley Scales of Infant Development (BSID). This included suspicions of motor, language or cognitive delays based on the Ages and Stages Questionnaire (ASQ). In some cases, diagnoses of “high-risk for cerebral palsy” were made based on clinical history, brain imaging and standardized neurologic exams using the Hammersmith Infant Neurologic Exam (HINE) administered virtually. Definitive diagnoses of cerebral palsy, however, were only made in person. We excluded children who did not attend an 18–24-month visit. Baseline characteristics, including sociodemographic characteristics (maternal age and caregiver education) and neonatal characteristics [birthweight (BW), GA, sex, small for gestational age (SGA) status defined as BW < 10th percentile for GA and sex, multiple gestations, Grade III or IV intraventricular hemorrhage (IVH), periventricular leukomalacia (PVL), severe ventriculomegaly > 15 mm on either side, bronchopulmonary dysplasia (BPD) and necrotizing enterocolitis (NEC) stage II or III] were collected retrospectively from electronic and paper hospital charts. These characteristics were collected for all infants who attended follow-up. Follow-up characteristics included GA at discharge home, presence of cerebral palsy (CP), BSID, 3rd or 4th edition composite scores in the motor, language and cognitive subtests, Ages and Stages Questionnaire, 3rd edition (ASQ-3) scores, and hearing and visual impairment. For the purposes of this study, CP included both suspected and definitive CP diagnoses.

The primary outcomes for the quantitative study were neurodevelopmental impairment (NDI) and significant neurodevelopmental impairment (SNI) at 18–24 months CA. Neurodevelopmental impairment was defined as a composite of neuromotor, neurocognitive and/or neurosensory impairment (Table 1) [15,16,17,18]. Cerebral palsy was defined as a non-progressive condition affecting movement and posture as a result of an early brain injury [19,20]. The severity of impairment was determined by the most significant impairment in any domain. For example, if a child was found to have CP with Gross Motor Functional Classification Scale (GMFCS) III, IV or V, BSID cognitive composite score 87, BSID language composite score 65 and normal hearing and vision, they would be classified as having SNI based on their GMFCS and their BSID language score. Children who could not be tested using the BSID but who had a BSID General Adaptive Composite score of <70 or were considered to have SNI. Secondary outcomes included the individual components of NDI and SNI. An additional variable, termed neurocognitive delay, was also added to assess cognitive and language development when in-person standardized testing was not available. This was defined as mild to moderate if the ASQ-3 communication, personal-social or problem-solving domains were 1–2 SD from the mean or if the BSID language or cognitive subtest scores were 1–2 SDs below the mean (i.e., scores between 70 and 85). Neurocognitive delay was considered significant if the ASQ communication, personal-social or problem-solving domains were <2 SD below the mean or if the BSID language or cognitive subtest scores were <2 SD below the mean (score < 70) [21]. If both an ASQ and a BSID were available, the BSID scores were used preferentially. Unfortunately, given the retrospective nature of this study, it was not possible to eliminate measurement bias introduced by using two different tools, one that is caregiver-reported and one that involves standardized testing conducted by a trained professional. In addition, the ASQ-3 has good sensitivity and specificity for the BSID [22] and can be used as an alternative to the BSID, albeit with some limitations in its predictive ability [23,24].

### 2.3. Data Analyses

#### 2.3.1. Qualitative Data Analysis

Descriptive analysis was used for baseline characteristics to contextualize findings. Interviews were transcribed verbatim and checked for accuracy using a professional transcription service. A thematic analysis was conducted according to Boyatzis et al. [8] to identify, analyze and report patterns or themes within the data. Thematic analysis occurred in the following stages—(1) an immersive reading of the data where initial ideas were noted in an iterative process; (2) generation of codes in a systematic fashion across the entire data set; (3) review of initial codes by the study group to ensure accuracy with feedback following initial coding used to enhance subsequent interviews; (4) collating codes into potential themes that were then further reviewed, defined and named in order to develop refined themes with clear definitions; and (5) relating the analysis back to the original theme. The same analysis approach was used for caregiver and healthcare provider interviews. The data from the transcribed interviews of the families and healthcare providers were coded separately and themes were triangulated from both these sources of data. Congruent and incongruent themes were assessed between the caregivers and healthcare providers. Internal validity and reliability were ensured using verification strategies such as checking and confirming the data through the use of digital recorders, and peer review. Peer debriefing was achieved by multiple committee meetings, which will involve an in-depth reading of the coding to ensure credibility. External validity was obtained through the use of purposeful sampling as a variety of experiences, perspectives, and social phenomena were included to increase the applicability of the findings to other settings. An audit trail was kept to ensure that the processes, decisions, and procedures of the study were documented and justified.

#### 2.3.2. Quantitative Data Analysis

##### Baseline and Follow-Up Characteristics

Descriptive statistics were used to compare baseline and follow-up characteristics of the “virtual” group and “in-person” group. Continuous variables were displayed as mean and standard deviation (SD) or median and inter-quartile range (IQR) for normally and non-normally distributed data, respectively, while categorical variables were displayed as counts and percentages. Student’s *t*-test and Wilcoxon Rank Sum tests were used for normally and non-normally distributed variables, respectively. Categorical variables were compared using Chi-square tests or Fisher exact tests, as appropriate. Children with missing data for any variable were excluded from analysis for that variable and denominators are provided in tables. It was assumed that data were missing at random, thereby not systematically biasing the results [25].

##### Assessment of Neurodevelopmental Outcomes

Multivariable logistic regression modelling was conducted to compare the primary and secondary neurodevelopmental outcomes. The covariates adjusted for in the multivariable analyses identified a priori included GA, SGA, sex, BPD and primary caregiver education, based on several predictive models and observational studies suggesting strong, independent associations between these factors and long-term neurodevelopment [26,27,28,29]. Adjusted odds ratios (aORs) and 95% confidence intervals (CIs) were estimated for NDI, SNI and all secondary outcomes. Children who were lost to follow-up or who did not have a complete data set for a particular outcome were excluded from that analysis. Baseline characteristics of those lost to follow-up compared to those who attended were compared. All statistical analyses were conducted using SAS software v.9.4 (SAS Institute Inc., Cary, NC, USA) with a 2-sided significance level of 0.05.

### 2.4. Sample Size Calculation

#### 2.4.1. Qualitative Study

For the qualitative part of the study, interviews were conducted until saturation was achieved in each theme. This was generally estimated to be between 5 and 30 [30].

#### 2.4.2. Quantitative Study

With post hoc analysis, assuming a baseline incidence of NDI of 45% [18], we are able to detect a 20% difference in NDI with an α of 0.05 and a power of 80%.

## 3. Results

Table 2 provides the baseline characteristics of the families interviewed as part of the qualitative study. For the qualitative study, 14 families with 19 children were interviewed. Most of these children (17/19) were preterm and 3 children (16%) were diagnosed with a developmental delay, including cognitive, language and/or motor delay.

### 3.1. Common Themes in Caregiver and Healthcare Provider Perspectives of Virtual Care in NNFU

Three themes emerged from the qualitative study of caregiver and healthcare provider perspectives (Table 3):

1. Caregivers and healthcare providers experienced increased confidence in “in-person” assessments compared to virtual assessments, related to the ability to perform physical examinations and interact with the child. In addition, communication was more effective “face to face”. Caregivers felt that the onus of identifying issues was less when the assessment was in-person. One difference between caregivers and healthcare providers was the healthcare provider’s perception that post-pandemic, patients were still opting for virtual appointments because of distance or because there were no concerns about the child’s development, while caregivers clearly stated that they would rather travel to an in-person appointment even in the absence of developmental concerns.
2. When “in-person” visits are not available or possible, virtual platforms can be used to deliver information. Caregivers were generally very grateful to have a virtual option during the pandemic and the ongoing availability of access to child development expertise. Healthcare providers similarly appreciated being able to offer their expertise during a time when these children could not be seen in person for those who may experience difficulty attending their in-person appointments. Interestingly, while caregivers felt that the virtual appointments worked quite well, healthcare providers felt that there were limitations in their scope of practice and that all required equipment was not available. In addition, recognizing the potential value of virtual care for specific situations, healthcare providers felt it was important to triage appropriate patients and families for virtual care. Healthcare providers consistently reported feeling inadequately prepared for virtual care delivery when the pandemic began given that for all the providers interviewed, this was their first time using virtual care in any clinical setting. Each clinician also reflected that given their different scopes of practice, issues that they were not as well-trained for in “in-person” clinical settings became even more limited in virtual settings. Effective communication using a virtual platform was also challenging.
3. Caregivers preferred the care provided at this specialized clinic for their child’s growth and development while healthcare providers felt that some patients could be followed at their primary care clinics for in-person assessments and supplemented by virtual assessments in a specialized clinic. Many caregivers noted inconsistent communication between the healthcare provider and family and wanted more visits in the NNFU. In addition, communication between the clinic and community providers was limited. On the other hand, healthcare providers felt that involving community providers would not necessarily be helpful during visits, although shared care models may address the gaps in access and limited resources of a large, tertiary specialized developmental clinic. Healthcare providers also felt that the information provided in follow-up emails or letters was largely sufficient.

### 3.2. Neurodevelopmental Outcomes with Virtual Care

**Figure 1:**
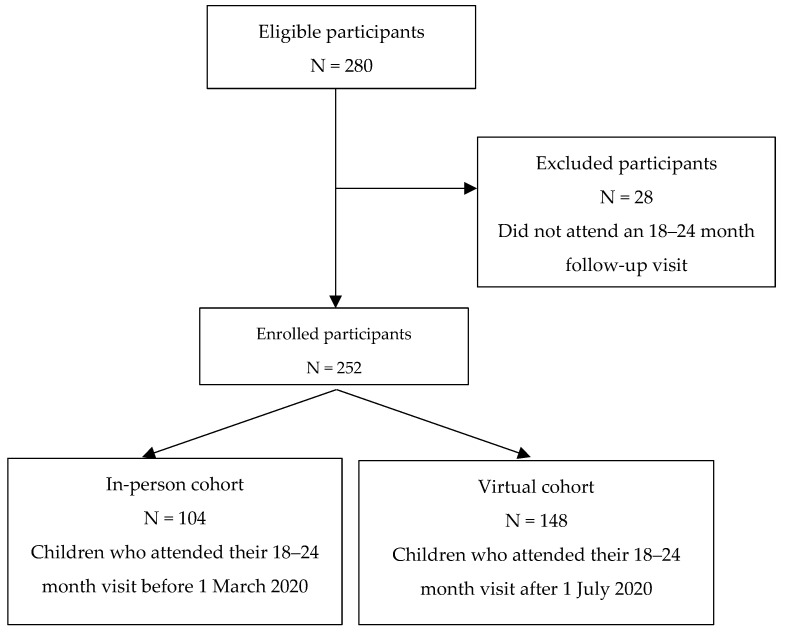
Flow diagram of participants in the quantitative study.

Figure 1 shows the flow diagram of participants in the quantitative study. Baseline characteristics are shown in Table 4. There were no differences found between children who were primarily followed after “virtual care” was implemented compared to those who were only seen “in-person”. Univariate analysis comparing follow-up characteristics, including motor, cognitive and language development, is presented in Table 4. A higher proportion (37% compared to 17%) of children in the virtual group were found to have BSID cognitive subtest scores <85 compared to those who were seen only “in-person”. Of note, 62/148 (42%) children completed at least part of the BSID in-person. Given that this was the minority of children, a separate neurocognitive outcome using the ASQ and BSID results together was also assessed. The overall proportion of children diagnosed with cerebral palsy was low, and there was a non-significant decrease in the virtual care group compared to the in-person group. Very few children were found to have hearing or vision impairment in both groups overall.

As loss to follow-up is up to 20% of children attending neonatal follow-up, Table S1 shows no significant differences in baseline characteristics between children who attended follow-up and those who were lost to follow-up except for maternal age, which is unlikely to be clinically significant.

Table 5 shows the neurodevelopmental outcomes of the two cohorts. There were no significant differences in NDI or SNI when multivariable logistic regression was used to account for confounding factors. The adjusted odds of cognitive delay measured by the BSID were significantly higher in the virtual group, while the adjusted odds of cerebral palsy were significantly lower in the virtual group. When caregiver-reported development using the ASQ was used in combination with BSID results, there was no longer a significant difference between the two cohorts in cognitive or language development.

## 4. Discussion

### 4.1. The Results of the Qualitative Interview of Parents and Healthcare Providers

This mixed-methods study included a qualitative analysis of caregiver experiences and healthcare provider perspectives and a quantitative analysis of long-term neurodevelopmental outcomes associated with the introduction of virtual care into the NNFU. Parents and caregivers alike expressed a strong preference for in-person assessments and felt that the hands-on approach was crucial to being able to assess the child thoroughly, simultaneously taking the onus of identifying concerns away from the parents and more effectively being able to address issues such as language barriers and subtle developmental delays. This remained the case even when there was a perceived benefit to caregivers of virtual care, including travel time. Similar findings have been noted in ambulatory clinics [31], including one pediatric diabetes clinic, where while parents were satisfied with virtual visits, only 56% expressed that it was as good as an in-person encounter [32,33].

Healthcare providers, on the other hand, felt that virtual care could be used more in clinical practice, if purposeful training was provided in all aspects of care, including administrative tasks (e.g., providing instructions to caregivers), history taking, performing a physical exam or developmental assessment and providing counselling. One particular issue that was highlighted was the need for language interpretation that appeared to be limited using virtual means and reflects the emerging recognition of digital inclusivity as a social determinant of health [34]. Our clinic is a large tertiary clinic serving a diverse population in Toronto, Canada, and in surrounding areas of Ontario, necessitating the use of interpretation services and culturally sensitive care practices. This has implications for how effective virtual care can and should be delivered to families and requires further exploration in future studies to ensure equitable access to services.

One way to reconcile healthcare worker and caregiver preferences is to integrate parent preference with health assessment results and create an algorithm for triaging patients. In general, caregivers reported that virtual visits were more successful with younger children and children with no perceived concerns. This allowed for continued access to specialty care when they were faced with barriers, such as distance to the clinic or illness. Regardless, the option to attend in person should be offered to families, especially when concerns are expressed at any stage of development.

### 4.2. The Results of the Neurodevelopmental Outcomes Before and After the Introduction of Virtual Care

One interesting finding was that during the pandemic, the diagnosis of CP decreased significantly. Studies evaluating the incidence of cerebral palsy have shown varying trends in the prevalence of cerebral palsy [35,36]. However, given that this cohort only spanned a 3-year period, it is unlikely that this decrease can be attributed to a true decrease in incidence, but rather a reduced ability to identify cerebral palsy as a result of the use of virtual care. This issue has been raised previously in the use of telehealth in the assessment of spasticity in older children [12]. This is particularly concerning in the context of the efficacy of early intervention in improving not only motor but also cognitive outcomes, in infants born preterm [37,38,39]. In addition, our results indicated an increased odds of cognitive delay. This could be because those who were seen in person in the clinic were children reported to have concerns by caregivers or healthcare providers or could be a reflection of the effect of the pandemic, including the lack of availability of early intervention services, inability to attend daycare or school and social isolation [40,41]. Alternatively, parent-reported measures may not detect more subtle cognitive difficulties as well as rigorous standardized testing, as has been demonstrated previously [24].

### 4.3. Strengths and Limitations

The strengths of this study are its mixed methods incorporating qualitative data with quantitative data to support the findings from interviews. This study builds upon existing literature focused on parents as the primary stakeholders in determining the best models of care for their children in neonatal follow-up clinics and further provides specific situations where virtual care may be more or less appropriate [42]. In addition, this study was a relatively large study, conducted using rigorous qualitative methodology, with representation from caregivers who experienced NNFU prior to the pandemic and those who were only enrolled during the pandemic.

The limitations of our study included bias from loss to follow-up, confounding from non-random assignment to virtual visits, differences in measurement tools, and the lack of power for several of the secondary outcomes. In particular, the impact of the pandemic itself versus the use of virtual care in assessments is difficult to separate and assess. Unfortunately, due to the limitations of virtual care, standardized testing using the BSID was not possible for all children and the neurocognitive delay composite outcome incorporated results from the ASQ parent-reported questionnaire The ASQ is concurrently valid with the BSID and is a good estimate of standardized testing, but has higher negative predictive value and low positive predictive value [22]. As such, this composite outcome should provide an estimate of the cognitive outcomes during the pandemic and concurrently also provide us with a sense of the value of parent-reported questionnaires in assessing cognitive development for preterm children. As with most studies involving neonatal neurodevelopmental follow-up, loss to follow-up may bias results. In order to assess the extent of the effect, we assessed the differences in maternal age, education level and common neonatal morbidities (Supplementary Materials, Material S1, Table S1) and noted a difference in maternal age with younger mothers more likely to be lost to follow-up. It is unclear if this is a clinically significant finding, and other factors were not found to be significant. However, it is possible that unassessed factors related to socioeconomic status resulted in poorer follow-up rates and there is a known impact of socioeconomic status compounding the effect of prematurity in long-term neurodevelopment [43,44]. Ultimately, larger prospective observational studies will be necessary to assess the impact of the pandemic on long-term neurodevelopment. Finally, it is possible that we included a less diverse population by excluding caregivers who were unable to converse in English for the interview.

## 5. Conclusions

In conclusion, virtual care has a valuable and likely permanent place in our medical system, improving access to services and enabling communication with other providers. A targeted triage process should be implemented in order to use virtual care optimally to ensure that conditions requiring in-person assessment are being identified while also allowing families to choose visit types that are ideal for them. Healthcare providers require focused training in order to use virtual care, ideally integrated with other digital means of communication (e.g., electronic handouts or social media). Finally, the potential impact of the pandemic on early development should be further evaluated and developmental services should be prioritized as essential services moving forward.

## Figures and Tables

**Table 1:** Definitions of neurodevelopmental impairment.

Impairment	Normal	NDI (Any One or More of the Following)	SNI (Any One or More of the Following)
Motor	Normal examination	CP with GMFCS 1 or higher	CP ^1^ with GMFCS 3, 4 or 5
		BSID score < 85	BSID score < 70
Cognitive	BSID score ≥ 85	BSID score < 85	BSID score < 70
Language	BSID score ≥ 85	BSID score < 85	BSID score < 70
Hearing ^2^	Normal	Sensorineural/mixed hearing loss	A hearing aid or cochlear implant
Vision ^3^	Normal	Unilateral or bilateral visual impairment	Bilateral visual impairment

BSID = Bayley Scales of Infant Development, 3rd of 4th edition; NDI = neurodevelopmental impairment; SNI = significant neurodevelopmental impairment. ^1^ Cerebral palsy defined as per Rosenbaum et al. [20]. ^2^ Hearing impairment determined from the most up-to-date audiology reports. ^3^ Visual function determined from the most up-to-date ophthalmology report. If no report is available, then impairment is defined as a small scarred eye, sustained sensory nystagmus or lack of response to a 1 cm object (cheerio) on a white background at 30 cm.

**Table 2:** Baseline characteristics of participating families.

	Interviewees [N = 14]
Maternal age (years), median (range)	35 (29, 39)
Gestational age (weeks), median (range)	29.7 (24.4, 40.1)
Birthweight (grams), median (range)	1200 (620, 3110)
Female sex, n (%)	13 (68)
SGA, n (%)	2 (11)
Multiples, n (%)	8 (42)
Significant brain injury ^1^, n (%)	1 (5)
Bronchopulmonary dysplasia, n (%)	4 (23)
Necrotizing enterocolitis, n (%)	0 (0)
Developmental delay, n (%)	3 (16)

% = percentage; n = number; SGA = small for gestational age. ^1^ including Grade III/IV intraventricular hemorrhage, periventricular leukomalacia and severe ventriculomegaly > 15 mm on either side.

**Table 3:** Caregiver and Healthcare Provider Perspectives of Virtual Care in Neonatal Neurodevelopmental Follow-up.

	Caregivers	Healthcare Providers	Congruent/ Incongruent?
Theme 1: Increased confidence in “in-person” assessments compared to virtual assessments	“I find “in-person” visits much more useful. I feel like if the goal of the visit is for a clinician to assess how the child and infant is doing, I think that this way you can actually touch and feel and look at and examine that child physically in person, as opposed to watching them on a screen…” “Virtual went okay. […] personally I tried to get into “in-person” because I just think, … I guess the onus was less on me to be like monitoring development.” “I always prefer “in-person”. I think in-person is better for assessment, observation and also communication…”	“Language barriers resulted in difficulty instructing caregivers effectively in how to use the technology, how to complete caregiver-reported questionnaires, how to perform parts of the physical exam and counselling around developmental activities.” “Forced to move to virtual visits…[and] there’s a missed opportunity to see kids who might be better served with an in-person visit” “I find that people still, even now, …are opting for virtual over in-person visits simply because of distance and…secondly because they have no concerns about their child”	Congruent
	Regarding virtual visits: “If there is one thing, I feel about is maybe the session is not long enough., it’s just in the clinic I had the feeling time was limited so we needed to go through a lot of things in a certain time.” Regarding in-person visits: “It was a little bit more rushed…I don’t know how the nurse or the doctor was really able to assess the kids.”		Neutral
	Regarding social media: “there are some pages that I follow on Instagram…if I am not getting [information] from Sinai or my pediatrician, … I think it would be even more positive because I’d know that the information that I’m provided is accurate.”	Regarding social media: “Using other virtual means, such as social media, could also enhance the care provided in clinic both virtually and in-person.” “[I] see a role for social media in providing educational platform, [but it] would be challenging with families where English is not the first language”	Congruent
	“the twins were not cooperating that morning and being at home alone with the two kids and trying to have a conversation, it just didn’t work”	“caregivers often joined visits while driving or distracted by other children or activities at home”	Congruent
Theme 2: When “in-person” visits are not available or possible, virtual platforms can be used to deliver information.	“given the times and the pandemic that we were in, I was very grateful to have [the visit] not cancelled, and that we were still able to pivot and do it virtually, so that I still had some information.”	“there’s a good role for virtual visits in our clinic. Because we are a tertiary centre, we have a caseload from vast parts of the province, and I think it’s very helpful to be able to offer virtual visit[s] for families who face barriers to travelling to Toronto or who have childcare barriers” “[the pandemic] showed that you can do virtual visits and they’re meaningful and helpful for the families.” “not the same as in-person, but lots of opportunity for coaching” “there has to be some triaging around who would benefit most from virtual care” [and] “coming up with an algorithm…[to understand] who is going to benefit [is important]”.	Congruent
	“in general [virtual appointments] actually worked better than I thought” and another said “Our... [virtual appointment] was fantastic even though we couldn’t be there in person. We still felt she did a fairly good assessment on [our child]”	“we didn’t have the appropriate equipment and/or the questionnaires” Regarding further limitations in scope of practice using virtual care: “my role…is to assess fine motor, not feeding necessarily unless it was oral motor challenges.” “no guidance or education [was provided] for virtual etiquette—[we] just jumped in” “I primarily sought out information on my own about using the platform…[and] I attended this online conference about virtual care”	Incongruent
Theme 3: Caregivers preferred the care provided at this specialized clinic for their child’s growth and development while healthcare providers felt that some patients could be followed at their primary care clinics for in-person assessments and supplemented by virtual assessments in a specialized clinic.	Described inconsistency amongst clinicians and the follow-up materials that were received “depending on clinician and visit”. In general, caregivers “appreciated continuity of care.” “I think a personalized email regarding the development of my babies with information related to their development was very useful”	“follow-up letters and emails were sent providing a report and resources”	Incongruent
	“would have appreciated less pediatrician visits and more clinic visits due to specialized knowledge.” “I don’t really see much connection between the clinic and the pediatrician.”	“some visits we could not do virtually, some of our assessments… [especially with] the older kiddies” “not sure if involving community providers would have been helpful for visits”	Incongruent

**Table 4:** Baseline and follow-up characteristics of participants in the quantitative study.

	Virtual (N = 148)	In-Person (N = 104)	*p*-Value
Maternal characteristics			
Maternal age (years), mean (SD)	33.07 (5.27)	32.55 (4.66)	0.42
Caregiver education (college+), % (n/N)	90 (75/83)	91 (84/92)	0.83
Neonatal characteristics			
Birth weight (g), median (IQR)	1025 (810, 1260)	990 (755, 1245)	0.49
Gestational age (week), median (IQR)	28.0 (25.5, 29.0)	28.0 (26.0, 29.5)	0.47
Sex (male), % (n/N)	50.7 (75/148)	60.0 (53/104)	0.96
SGA, % (n/N)	16 (23/148)	22 (23/104)	0.18
Multiple gestations, % (n/N)	30 (44/148)	28 (29/104)	0.75
Grade III/IV IVH, % (n/N)	4 (6/147)	8 (8/100)	0.19
BPD at 36 weeks PMA, % (n/N)	44 (60/137)	57 (52/92)	0.059
Follow-up Characteristics			
GA at discharge home, median (IQR)	34 (32, 37)	34 (32, 37)	0.64
BSID motor composite score < 85, % (n/N)	21 (11/53)	12 (10/85)	0.15
BSID cognitive composite score < 85, % (n/N)	37 (23/62)	18 (17/97)	**0.006**
BSID language composite score < 85, % (n/N)	44 (23/52)	35 (29/83)	0.28
Neurocognitive delay, % (n/N)	25 (37/148)	17 (18/104)	0.15
Significant neurocognitive delay, % (n/N)	16 (23/148)	15 (16/104)	0.97

Notes: The reported *p*-values were based on the comparisons between two groups using the Chi-square test for categorical variables and the Student’s *t*-test or Wilcoxon rank sum test as appropriate for continuous variables. % = percentage; BPD = bronchopulmonary dysplasia; IQR = interquartile range; n = number; N = available data; PMA = postmenstrual age; SD = standard deviation; SGA = small for gestational age. There were no significant differences between groups in the rates of periventricular leukomalacia, severe ventriculomegaly, necrotizing enterocolitis, cerebral palsy, BSID cognitive, language or motor score < 70 or ASQ scores < 1 SD or <2 SD from the mean, hearing loss requiring amplification or bilateral visual impairment. Bolded = *p* < 0.05.

**Table 5:** Neurodevelopmental outcomes of participants attending follow-up.

	Virtual % (n/N)	In-Person % (n/N)	OR (95% CI)	AOR * (95% CI)
Primary outcomes				
SNI	10 (14/140)	12 (12/97)	0.79 (0.35, 1.78)	0.97 (0.38, 2.48)
NDI	48 (67/140)	68 (67/98)	0.42 (0.25, 0.73)	0.85 (0.44, 1.66)
Secondary outcomes				
Composite motor score < 85	21 (11/53)	12 (10/85)	1.96 (0.77, 5.01)	2.10 (0.75, 5.88)
Composite cognitive score < 85	37 (23/62)	18 (17/97)	2.78 (1.33, 5.78)	**2.78 (1.25, 6.19)**
Composite language score, 85	44 (23/52)	35 (29/83)	1.48 (0.73, 3.00)	2.21 (0.99, 4.95)
Composite motor score < 70	6 (3/53)	4 (3/85)	1.64 (0.32, 8.44)	1.80 (0.34, 9.55)
Composite cognitive score < 70	8 (5/62)	4 (4/97)	2.04 (0.53, 7.91)	1.49 (0.26, 8.48)
Composite language score, 70	17 (9/52)	12 (10/83)	1.53 (0.58, 4.06)	1.46 (0.51, 4.21)
Cerebral palsy	2 (2/120)	7 (7/104)	0.23 (0.05, 1.16)	**0.11 (0.01, 0.98)**
Mild-moderate neurocognitive delay	25 (37/148)	17 (18/104)	1.59 (0.85, 2.99)	1.99 (0.94, 4.23)
Significant neurocognitive delay	16 (23/148)	15 (16/104)	1.01 (0.51, 2.03)	0.83 (0.35, 1.98)

Notes: * AOR = adjusted OR, based on the multiple logistic regression model adjusted for potential confounders including BPD at 36 weeks, sex, SGA, GA and education. % = percentage; n = number. Bolded = *p* < 0.05.

## Data Availability

The data presented in this study are available on request from the corresponding author. The data are not publicly available due to privacy.

## Supplementary Materials

The following supporting information can be downloaded at: https://www.mdpi.com/article/10.3390/children11111272/s1, Table S1: Baseline characteristics of participants who were lost to follow-up; Material S1: Interview questions.
