# Supplementary material for: Caregiver Experiences, Healthcare Provider Perspectives and Child Outcomes with Virtual Care in a Neonatal Neurodevelopmental Follow-Up Clinic: A Mixed-Methods Study
Source: Children (Basel). 2024 Oct 22;11(11):1272. doi: 10.3390/children11111272 (PMC11592627; doi:10.3390/children11111272)
Supplement: Supplementary file 1 [file children-11-01272-s001.zip › children-3202130-supplementary.pdf]

Table S1: Baseline characteristics of participants who were lost to follow-up.

	**Follow-up (N=252)**	**Lost to follow-up (N=28)**		**P value**
**Maternal characteristics**				
Maternal age (years), mean (SD)	32.86 (5.03)	30.46 (4.50)		0.02
Caregiver 1 education (college+), % (n/N)	91 (159/175)	100 (2/2)		1.00*
**Neonatal characteristics**				
Birth weight (g), median (IQR)	1005 (790, 1250)	1060 (845, 1505)		0.30
Gestational age (week), median (IQR)	28.0 (26.0, 29.0)	28.0 (25.5, 31.0)		0.71
Sex (male), % (n/N)	51 (128/252)	50 (14/28)		0.94
SGA, % (n/N)	18 (46/252)	14 (4/28)		0.60
Grade III/IV IVH or PVL, % (n/N)	6 (14/247)	4 (1/28)		1.00*

Notes: the reported p-values were based on the comparisons between two groups using the Chi-square or * Fischer exact test as appropriate for categorical variables and the Student t test or Wilcoxon rank sum test as appropriate for continuous variables. % = percentage; BPD = bronchopulmonary dysplasia; IQR = interquartile range; n = number; PDA = patent ductus arteriosus; SD = standard deviation; SGA = small for gestational age. There were no significant differences noted in severe brain injury, including Grade III/IV intraventricular hemorrhage, periventricular leukomalacia and severe ventriculomegaly > 15 mm on either side. There was also no significant difference in necrotizing enterocolitis, stage II-III

Material S1

Interview Questions

I. Patient/Family Interview

For families who received both in-person and virtual visits:

General questions:

1. Please tell us what your overall experience has been with our clinic, including virtual and in-person visits.
2. Can you tell us a little about your child? How are they doing?
3. Could you tell us how many virtual visits you had and when? And how many in-person visits you've had and when?
   For interviewer: visits occur at 3 months, 8 months, 12 months, 22 months and 3 years. All visits are corrected age except 3 years. There is a speech group session between 15-18 months virtually.

Development-specific questions:

1. During your visits, was feeding discussed or assessed? How did that occur over virtual platforms? Are there aspects that could be improved for the future?
2. How were you able to provide a weight measurement? Did you have access to body measurements during the pandemic? For example, do you have a baby weighing scale at home that you can use?
3. What kind of coaching/counseling regarding motor development did you get? (for example, did you have someone talk to you about tummy time). Are there other ways in which we could have provided this information (i.e. using a video or using a prop like a doll, etc., posting information on social media or a follow-up website?)
4. Did you want any support for mental health (for example, if you were feeling anxious or if you were unhappy or had low mood) after your baby was discharged from the hospital? If you did, was this available to you and easy to access? Was this provided virtually or in-person? Would regular information sessions about parent mental health have been helpful to you? How about social media or peer group connections?
5. Did you have specific behavioural concerns for your child? For example, sleep or tantrums. How were these addressed? Did you receive support for your child's behavioural challenges? Was this helpful? How might this be improved?
6. Do you have other community providers involved (e.g. occupational therapist, physiotherapist, infant development etc.)? How might we have better involved your community providers in virtual visits? Would their involvement have been helpful to you in this process?
7. Was autism spectrum disorder ever discussed with you during your visits? Were you aware that screening for ASD is conducted routinely in our clinic?
   a. If child had red flags for autism/diagnosed with autism: How were concerns over social communication discussed with you? What happened when these concerns were identified? Were additional, specific resources sent to you to discuss this issue?

Types of Care Platforms

1. Can you comment on the types of virtual care you have experienced in our clinic (i.e. telephone vs Zoom vs other)?
   a. If there was correspondence sent after the visit, how did you find this messaging? What platform was it done with (i.e. texting vs email etc.)? Would you have liked more messaging? Were local resources part of this information?

2. Can you comment on how you think the assessment went using virtual care? Were there specific parts of the physical examination, exercises or counselling that were particularly challenging or needed improvement?
3. Would another mode of visit have been more appropriate (i.e. in-person, telephone or other)?
4. Do you see a role for social media in providing educational content? Would you have liked to use social media to get some of this information? (e.g. Instagram or twitter)

Digital Privacy and Consent

1. Did you feel like your privacy was respected in this process? How might we improve privacy?
2. Prior to starting a virtual visit, was there specific communication regarding your agreement to participate and the risks of using virtual media platforms?

Equipment

1. What type of technology do you have access to (i.e. computer vs tablet vs smartphones)?
    a. How did you find its technical quality for the purpose of a clinical visit?
    b. Did you feel supported during the visit from a technical standpoint? That is, were we able to help you troubleshoot when technology was not working?

Virtual Care Workflow

1. How did scheduling occur at your virtual visit? At what point did you have to schedule the visit?
2. How were you informed of your appointment? Was communication clear as to when the appointment was and how it would be conducted?
3. Did you find the scheduling easy or was it a challenge to find a mutually beneficial time? Was this different in any way from in-person visits?
4. Did you receive documentation relevant to the visit? Were you able to understand this documentation? How did you receive it? Was it helpful? How could this be improved?

Patient Setup and Education

1. Were you able to effectively use the chosen platform? Was there enough support to use that platform? Did you experience any technical issues?

Etiquette

1. Did you feel that the staff member was easily visible on the video platform? (or easily audible on the audio platform)?
2. Were you able to understand the information provided or the questions asked during the visit?
3. Were you given clear and concise instructions for the assessment?

Environment and setting

1. Did you have a private space to complete the virtual visit with the ability for your child to move?
2. Did you know/recognize all members on the video platform? Do you feel like it would have been helpful to see the same provider at each visit?
3. Did you feel like giving the provider a chance to see your home helped understand some of the concerns you had or challenges you faced?
4. Were you able to use a high-definition camera? Were there issues with not being able to see important parts of the assessment?
5. Were there issues with the internet quality?

Conclusion

1. Any last thoughts about the use of virtual care in our clinic?
2. Any suggestions for us going forward if virtual care were to be integrated into routine practice?

For families who received only in-person visits:

General questions:

1. Please tell us what your overall experience has been with our clinic, including virtual and in-person visits.
2. Can you tell us a little about your child? How are they doing?

Development-specific questions:

1. During your visits, was feeding discussed or assessed? How did that occur? Are there aspects that could be improved for the future?
2. What kind of coaching/counseling regarding motor development did you get? (for example, did you have someone talk to you about tummy time). Are there other ways in which we could have provided this information (i.e. using a video or using a prop like a doll, etc., posting information on social media or a follow-up website?)
3. Did you want any support for mental health (for example, if you were feeling anxious or if you were unhappy or had low mood) after your baby was discharged from the hospital? If you did, was this available to you and easy to access? Was this provided virtually or in-person? Would regular information sessions about parent mental health have been helpful to you? How about social media or peer group connections?
4. Did you have specific behavioural concerns for your child? For example, sleep or tantrums. How were these addressed? Did you receive support for your child's behavioural challenges? Was this helpful? How might this be improved?
5. Do you have other community providers involved (e.g. occupational therapist, physiotherapist, infant development etc.)? How might we have better involved your community providers in virtual visits? Would their involvement have been helpful to you in this process?
6. Was autism spectrum disorder ever discussed with you during your visits? Were you aware that screening for ASD is conducted routinely in our clinic?
    a. If child had red flags for autism/diagnosed with autism: How were concerns over social communication discussed with you? What happened when these concerns were identified? Were additional, specific resources sent to you to discuss this issue?

Types of Care Platforms

1. Was correspondence sent after the visit? How did you find this messaging? What platform was it done with (i.e. texting vs email etc.)? Would you have liked more messaging? Were local resources part of this information?
2. Can you comment on how you think the assessment went in-person? Were there specific parts of the physical examination, exercises or counselling that were particularly challenging or needed improvement?
3. Would another mode of visit have been more appropriate or convenient (i.e. virtual video, telephone or other)?
4. Do you see a role for social media in providing educational content? Would you have liked to use social media to get some of this information? (e.g. Instagram or twitter)

Privacy and Consent

1. Did you feel like your privacy was respected in this process? How might we improve privacy?
2. Can you comment on the consent process used for your in-person visit? (i.e. form vs verbal vs other vs none)

Workflow

1. How did scheduling occur at your virtual visit? At what point did you have to schedule the visit?
2. How were you informed of your appointment? Was communication clear as to when the appointment was and how it would be conducted?
3. Did you find the scheduling easy or was it a challenge to find a mutually beneficial time?
4. Did you receive documentation relevant to the visit? Were you able to understand this documentation? How did you receive it? Was it helpful? How could this be improved?

Etiquette

1. Were you able to understand the information provided or the questions asked during the visit?
2. Were you given clear instructions for parts of your visit?

Environment and setting

1. Did you have a private space to complete the visit with the ability for your child to move?
2. Did you know/recognize all members at your visit?
3. Do you feel like giving the provider a chance to see your home would have helped understand some of the concerns you had or challenges you faced?

Conclusion

1. Do you have any thoughts about the way in-person visits are conducted? Any suggestions for us going forward if virtual care were to be integrated into routine practice?

II. Healthcare Provider Interview

General questions:

1. Please tell us what your overall experience has been with our clinic, including virtual and in-person visits.
2. Can you tell us a little about your practice? How long have you been in this position? Have you had previous experience in a similar clinic? Have you done any work in virtual care before?

Development-specific questions:

1. During your visits, was feeding discussed or assessed? How did that occur over virtual platforms? Are there aspects that could be improved for the future?
2. How were you able to obtain a weight measurement? Did you have access to the child's body measurements from outside the clinic?
3. Was there adequate coaching/counseling regarding motor development? (i.e. tummy time etc.). Are there other ways in which we could have provided this information (i.e. using a video? Posting information on social media or a follow-up website?)
4. Did we provide any social support virtually? Would regular information sessions and/or social support have been helpful?
5. Was support for behavioural challenges provided? Was this helpful to the family? How might this be improved?

6. How might we have better-involved community providers in virtual visits? Would their involvement have been helpful to you and to the family in this process?
7. It has been reported that there is less support around social communication and language resources. Did you feel like this was the case? Was autism spectrum disorder ever discussed during visits?
    a. If child had red flags for autism/diagnosed with autism: How were concerns over social communication discussed? Were additional, specific resources sent regarding this issue?

Types of virtual care platforms

1. Can you comment on the types of virtual care you used in our clinic (i.e. telephone vs Zoom vs other)? Were these visits done from home or from the clinic?
    a. If there was correspondence sent after the visit, how did you find this messaging? What platform was it done with (i.e. texting vs email etc.)? Were local resources part of this information?
2. Can you comment on specific aspects of the visit using virtual care (e.g. physical examination, test results, counselling, treatments, prognosis etc.)?
3. Would another mode(s) of visit have been more appropriate (i.e. in-person, telephone or other)?
4. Do you see a role for social media in providing educational content? Would you have liked to use social media to get some of this information? (e.g. Instagram or twitter)

Digital Privacy and Consent

1. Did you feel like your privacy was respected in this process? How might we improve privacy?
2. Can you comment on the consent process used for your virtual visit? (i.e. form vs verbal vs other vs none)

Equipment

1. What type of technology do you have access to (i.e. computer vs tablet vs smartphones)?
    a. How did you find its technical quality for the purpose of a clinical visit?
    b. Did you feel supported during the visit from a technical standpoint? That is, were we able to help you troubleshoot when technology was not working?

Virtual Care Workflow

1. How was the family informed of their appointment? Was communication clear as to when the appointment was and how it would be conducted?
2. Did you find the scheduling easy or was it a challenge to find a mutually beneficial time? Was this different in any way from in-person visits?
3. Did you or someone from the clinic send documentation relevant to the visit? Were families able to understand this documentation? How was it sent? Was it helpful? How could this be improved?

Patient Setup and Education

1. Were you able to effectively use the chosen platform? Was there enough support to use that platform? Did you experience any technical issues?

Etiquette

1. Did you feel that you were easily visible on the video platform? (or easily audible on the audio platform)?
2. Were you able to communicate important information during the visit?
3. Were clear instructions for things like physical exam maneuvers or play items provided? How were these communicated?

4. How did you convey compassion during these virtual visits?
5. Was there guidance or education provided prior to the start of the transition to virtual care in terms of how best to use the technology and what the expectations were with regard to etiquette?

Environment and setting

1. Did you have a private space to complete the virtual visit?
2. Did you feel like getting a chance to see the family's home environment helped understand some of the concerns they had or challenges they faced?
3. Were you able to use a high-definition camera? Were there issues with not being able to see important parts of the assessment?
4. Were there issues with the internet quality?

Conclusion

1. Any last thoughts about the use of virtual care in our clinic?
2. Any suggestions for us going forward if virtual care were to be integrated into routine practice?
